# Correlation effect of EGFR and CXCR4 and CCR7 chemokine receptors in predicting breast cancer metastasis and prognosis

**DOI:** 10.1186/1756-9966-29-16

**Published:** 2010-02-24

**Authors:** Yixin Liu, Ru Ji, Jingyong Li, Qiang Gu, Xiulan Zhao, Tao Sun, Jinjing Wang, Jiangbo Li, Qiuyue Du, Baocun Sun

**Affiliations:** 1 Department of Pathology, Tianjin Medical University, Tianjin, China; 2 Department of Pathology, Tianjin Cancer Hospital, Tianjin Medical University, Tianjin, China; 3 The College of Public Health, Tianjin Medical University, Tianjin, China

## Abstract

**Background:**

The chemokine receptors CXCR4 and CCR7 play an important role in cancer invasion and metastasis. This study investigated the expression of CXCR4, CCR7, CXCL12, CCL21, and EGFR to illustrate the role of these biomarkers in breast cancer metastasis and prognosis.

**Methods:**

The CXCR4, CCR7, CXCL12, CCL21, and EGFR biomarkers were analyzed along with ER, PR, and HER-2/neu in breast cancer tissue microarray (TMA) specimens, including 200 primary breast cancer specimens by immunohistochemistry. Corresponding lymph nodes from the same patients were also examined using the same method.

**Results:**

Together with their CXCL12 and CCL21 ligands, CXCR4 and CCR7 were significantly highly expressed in tumor cells with lymph node (LN) metastasis. Similarly, EGFR was expressed highly in tumors with LN metastasis. The ligands were especially expressed in metastatic tumors than in primary tumors from the same patients. Moreover, the expression of both CXCR4 accompanied by CCR7 and CXCL12 accompanied by CCL21 were up-regulated. Kaplan-Meier survival analysis revealed that patients exhibiting high CXCR4, CCR7, and EGFR expression experienced a shorter survival period compared with those with low expression.

**Conclusions:**

The expression of CXCR4, CCR7, and EGFR may be associated with LN metastasis. Moreover, the expression of these receptors can serve as an indicator of undesirable prognosis in patients with breast cancer.

## Background

Breast cancer ranks among the most common malignant tumors afflicting women worldwide. Despite decreased mortality rates resulting from combined therapy, breast cancer remains a leading cause of cancer death in women. Particularly in the last two decades, incidence and mortality rates of breast cancer have climbed sharply in China, thus attracting increased attention from researchers.

Metastasis is one characteristic of malignant tumors which determines the course of therapy and cancer prognosis. It is a multifactorial, nonrandom, and sequential process with an organ-selective characteristic. In essence, axillary lymph node metastasis is the most frequently occurring metastatic disease; it can be seen as a surrogate for distant metastasis and long-term survival [1].

Although several molecules are involved in breast cancer metastasis, precise mechanism of tumor cell migration to specific organs remains to be established [2]. Previously, the "seed and soil" theory was employed to explain directional metastasis, considering that certain metastasis organs possess the congenial environment of the primary organ [3]. More recently, a "chemokine-receptor" model has been proposed to explain the homing of tumor cells to specific organs [4]. Chemokines belong to a super-family of small, cytokine-like proteins that induce cytoskeletal rearrangement and adhesion to endothelial and directional migration through their interaction with G-protein-coupled receptors [2,5].

Among the chemokines, the most interesting chemokine-receptor pair is the CXC chemokine receptor-4 (CXCR4) and its lone ligand, CXC chemokine ligand-12 (CXCL12). Muller demonstrated that CXCR4 is consistently expressed in human breast cancer cells, malignant breast tumor and metastasis tumors, while its ligand CXCL12 is preferentially expressed in the lungs, liver, bone marrow, and lymph nodes [2]. Thus, it can be deduced that the CXCL12-CXCR4 axis may be associated with the metastasis of breast cancer cells to the lungs, liver, bone, and lymph nodes. Unlike CXCL12, however, CC chemokine ligand-21 (CCL21) - the ligand for CC chemokine receptor-7 (CCR7) - is highly expressed in the lymph nodes of breast cancer patients [5]. Thus, the CCR7-CCL21 axis can be said to assume an important role in lymph node metastasis [6]. In this study, the expression of both CXCR4 and CCR7 is combined to evaluate their contribution in the lymph node metastasis of breast cancer. The importance of growth factors such as epidermal growth factor receptor (EGFR) and human epidermal growth factor receptor2 (HER-2/neu) has been established in the prognosis of breast cancer. Recently, several studies have revealed the crosstalk between CXCR4 and EGFR or HER-2/neu through transactivation by the CXCL12-CXCR4 axis.

This study aims to verify the significance of CXCR4, CCR7 and their CXCL12 and CCL21 ligands, together with EGFR in the evaluation of metastasis and the prognosis of breast cancer.

## Methods

### Patient selection and clinical data

The study group was composed of 200 specimens selected from 284 cases (84 cases were excluded owing to the absence of follow-up status) of female primary invasive duct breast cancer cases diagnosed between January 1997 and December 2004 at the General Hospital of Tianjin Medical University. Patients' records were retrieved and clinical data, histopathological record, and treatment information were all reviewed. All patients had not been subjected to chemotherapy and radiotherapy prior to surgical resection but had received chemotherapy following surgical operation. Follow-up information from all the patients were obtained by the authors themselves in August 2009 through visits or telephone interviews with either the patients or their relatives. Mean follow-up time was 88 months, ranging from 5 to 150 months. Formalin-fixed paraffin-embedded tumor materials and their lymph node tissues were acquired from the Department of Pathology of Tianjin Medical University's General Hospital. Tumor diameter, pathologic stage, and nodal status were selected from the primary pathology reports. All slides were reviewed by two pathologists to define histological types and grades.

### Construction of tissue microarray

Tissue microarray (TMA) blocks were constructed from formalin-fixed, paraffin-embedded breast cancer samples stored at the Department of Pathology of Tianjin General Hospital. These TMA blocks were composed of 200 paired samples (primary tumors and corresponding lymph nodes, either metastatic or non-metastatic). Haematoxylin and eosin stained slides were reviewed to confirm the diagnosis of invasive breast cancer; afterwards, two representative tumor regions were selected and marked on the donor blocks.

Tumor TMA blocks were created by punching a cylinder using a hollow needle with a diameter of 2 mm; the blocks were obtained from the two selected areas of each donor block before being inserted into an empty paraffin block. Subsequently, these blocks were cut into 4 μm thick slides and prepared for immunohistochemical (IHC) analysis.

### Immunohistochemical analysis

Using IHC staining, the expression of different proteins in human breast cancer was verified. In this process, sections were deparaffinaged in xylene prior to rehydration using gradient alcohol. Endogenous peroxydase activity was blocked by 3% hydrogen peroxide in 50% methanol for 20 minutes. For antigen retrieval, sections were treated with citrate buffer saline (pH 6.0) for 15 minutes at 95°C in a microwave oven. After blocking with 10% normal goat serum for 30 minutes at room temperature, sections were incubated with primary antibodies for another 30 minutes at room temperature. The sections were subsequently incubated for 16 hours at 4°C. Primary antibodies and dilution were as follows: rabbit polyclonal anti-CXCR4 (Abcam, dilution 1:100); rabbit polyclonal anti-CCR7 (Abcam, dilution 1:100); rabbit polyclonal anti-CXCL12 (Abcam, dilution1:100); goat polyclonal anti-CCL21 (Santa Cruz Biotechnology, dilution 1:50); rabbit polyclonal anti-EGFR (Santa Cruz Biotechnology, dilution 1:100); mouse monoclonal anti-ER (Zhongshan; ready-to-use); rabbit polyclonal anti-PR (Santa Cruz Biotechnology, dilution 1:100); and mouse monoclonal anti-HER2 (Zhongshan; ready-to-use).

Following incubation, sections were lavaged with phosphate buffered solution (PBS) and incubated with horseradish peroxidase (HRP)-conjugated goat anti-rabbit IgG, goat anti-mouse IgG, or rabbit anti-goat IgG for 40 min at room temperature. Staining was performed using 3,3'-diaminobenzidine (DAB). Sections were counterstained with haematoxylin followed by dehydration and mounting. Negative controls were prepared using PBS in lieu of the first antibody.

### Scoring of immunostaining

Sections were read by two separate pathologists blinded to patients' clinical pathology parameters. Both intensity and percentage of positive cells were considered. Five microscopy fields were reviewed in each core with 400× magnification, after which positive cells of 100 tumor cells in each field were counted. In staining for CXCR4, CCR7, CXCL12, CCL21 and EGFR, tumor cells with brown cytoplasm and/or nucleus or membrane were considered positive and then scored based on four classes: none (0); weak brown (1+); moderate brown (2+); and strong brown (3+). Percentage of stained tumor cells was categorized into five classes: 0 for negative cells, 1 for 1-25%; 2 for 25-50%; 3 for 50-75%; and 4 for >75%. Multiplication (staining index) of intensity and percentage scores was utilized to determine the result.

A staining index of ≥6 was defined as high expression, while <6 was defined as low expression [7]. On the another hand, HER2/neu was evaluated as positive when over 10% of tumor cells exhibited stained consecutive membranes. Unified standards were employed when evaluating estrogen receptors (ERs) and Progesterone receptors (PRs) that exceeded 10% of tumor cells, as shown in the stained nucleus.

### Statistical analysis

Analyses were performed using the SPSS 17.0 software package (Chicago, IL, USA). The relation between CXCR4, CCR7, EGFR, and clinicopathologic characteristics were tested via Pearson χ^2 ^analysis. The same method was employed to test associations between these biomarkers and biologic-prognostic characteristics, such as ER, PR, and HER-2/neu expression. Correlations between two variables were evaluated by Spearman's rank correlation test. P-values < 0.05 were deemed statistically significant. Overall survival (OS) was estimated through the Kaplan-Meier method and was compared between groups through the log-rank test.

## Results

### Characteristics of patients and expression of biomarkers in primary tumors

Patient and primary tumor characteristics are presented in Table 1. Samples included 200 patients, among which 100 developed lymph node metastasis while 100 did not. Median age was determined at 51 years (37-74). Thirty-nine patients (19.5%) were diagnosed with stage I cancer, 138 (69%) with stage II, 20 (10%) with stage III, and three (1.5%) with stage IV.

**Table 1:** Correlation between biomarkers and primary tumor characteristics

	CXCR4 cytoplasmic expression			CXCR4 nuclear expression			CCR7 expression			EGFR expression		
				
	Low	High	P	Low	High	P	Low	High	P	Low	High	P
	(n)	(n)		(n)	(n)		(n)	(n)		(n)	(n)	
age			.842			.409			.169			.299
<50	43	51		38	56		37	57		49	45	
≥50	47	59		49	57		52	54		63	43	
tumor size			.539			.106			.945			.525
D≤2	27	41		36	32		31	37		38	30	
2<D≤5	50	56		39	67		46	60		62	44	
D>5	13	13		12	14		12	14		12	14	
grade			.068			.985			.786			.030*
I	6	8		6	8		6	8		9	5	
II	59	73		58	74		61	71		81	51	
III	25	29		23	31		22	32		22	32	
stage			.148			.052			.086			.088
I	22	17		23	16		23	16		22	17	
II	61	77		58	80		60	78		82	56	
III	7	13		6	14		5	15		8	12	
IV	0	3		0	3		1	2		0	3	
LN			<.001**			.199			<.001**			.046*
negative	59	41		48	52		59	41		63	37	
positive	31	69		39	61		30	70		49	51	
N			.437			.534			.341			.770
N≤3	11	30		18	23		10	31		21	20	
3<N≤10	11	16		11	16		11	16		14	13	
N>10	9	23		10	22		9	23		14	18	
ER			.256			.117			.319			.087
negative	49	51		49	51		48	52		50	50	
positive	41	59		38	62		41	59		62	38	
PR			.115			.084			.249			.466
negative	51	50		50	51		49	52		54	47	
positive	39	60		37	62		40	59		58	41	
HER2/neu			.039*			.852			.099			.005**
negative	75	78		66	26		73	80		94	59	
positive	15	32		21	87		16	31		18	29	

D represents the diameter of tumor, LN represents lymph node status, N represents the amoumt of lymph node excised, grade means the histological grade, and stage means the clinical stage. *P < 0.05, **P < 0.01

**Figure 1:**
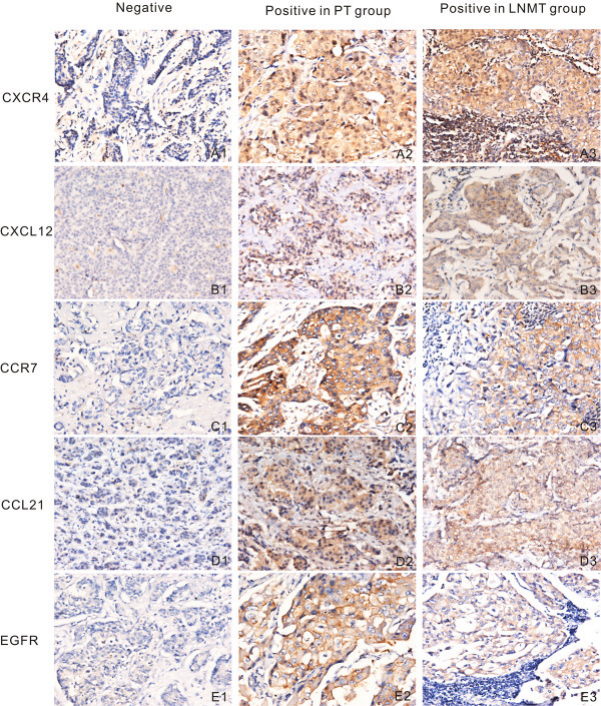
**IHC staining for biomarkers**. IHC staining for CXCR4, CXCL12, CCR7, CCL21 and EGFR. PT pertains to primary tumor, while LNMT stands for lymph node metastasis tumor. Rows correspond to the designated chemokine or receptor. The first column represents staining of negative expression in primary breast cancer with the indicated antibody. The second column indicates positive expression in primary breast cancer, and the third column shows positive expression in lymph node metastasis cancer. Both PT and LNMT columns in each row are obtained from the same patient while the negative column is not. In the CXCR4 row, A2 and A3 exhibit high expression in both cytoplasm and nucleus. CCR7, CXCL12, and CCL21 all exhibit positive reaction in the cytoplasm. In the EGFR row, E2 and E3 indicate that EGFR is expressed mainly in the membrane. However, a number of tumor cells appear to be positive in the cytoplasm as well (Panels A-E, ×200).

In IHC staining, 77% of tumor cells were CXCR4 positive in the cytoplasm, including high and low CXCR4 expression (Figure 1A2). Meanwhile, 73% were positive in the nucleus (Figure 1A2). The amounts of CCR7 (Figure 1B2) and EGFR (Figure 1E2) were detected in 82% and 66% of tumor cells, respectively, in the cytoplasm and/or membrane. Furthermore, 50% of ER, 49.5% of PR, and 23.5% of HER-2/neu were observed to be positive.

### Association of CXCR4, CCR7, and EGFR with lymph node metastasis

The immunoreactivity of CXCR4 was observed in the cytoplasm and/or nucleus of tumor cells. Cytoplasmic reactivity of CXCR4 correlated positively with lymph node metastasis of breast cancer (P < 0.001), but not with the amount of involved lymph nodes. Nuclear reactivity was not observed to be correlated with any pathologic parameters. Meanwhile, CCR7 was positively expressed in the cytoplasm, and the activity was significantly correlated with lymph node metastasis (P < 0.001). Similarly, associations among the lymph node status, histological grade, and EGFR expression were observed in this study (Table 1).

To verify the important effect of CXCR4 and CCR7 in metastasis, CXCR4, CCR7, and EGFR expression in primary breast cancer were compared with that in lymph node metastasis tumor. It was observed that CXCR4 and CCR7 expression in metastasis tumor was even higher, although no significant distinction was evident. More importantly, their respective ligands, CXCL12 and CCL21, exhibited significant differences in expression between primary tumor and lymph node metastasis tumor (P = 0.016 and P = 0.004; Table 2). Distinction between chemokines and their receptors with regard to distribution may be associated with the mechanism of metastasis; specifically, chemokines attract their receptors to certain sites along the chemokine concentration gradient.

**Table 2:** Differences of biomarkers between primary tumor and lymph node metastasis tumor

	cytoplasmic CXCR4			CCR7			CXCL12			CCL21			EGFR		
					
	Low	High	P	Low	High	P	Low	High	P	Low	High	P	Low	High	P
	(n)	(n)		(n)	(n)		(n)	(n)		(n)	(n)		(n)	(n)	
PT	31	69	.372	30	70	.336	62	38	.016*	52	48	.004**	49	51	.572
MT	38	62		23	77		45	55		32	68		53	47	

PT means primary tumor, MT means lymph node metastasis tumor. The differences of the biomarker between primary tumors and metastasis tumors were tested by pearson χ^2 ^analysis. *P < 0.05, **P < 0.01

### Correlation between CXCR4, CCR7, EGFR and HER-2/neu

Although neither ER nor PR positivity was associated with degree of the biomarkers, HER2 over-expression was correlated with CXCR4 cytoplasmic positivity (p = 0.039; Table 1). As indicated by reports, the expression rate of HER2/nu in breast cancer is approximately 25%. In the results of this study, the expression of HER2 was nearly 20%, and among CXCR4 cytoplasmic positive patients, approximately 40% were associated with HER2 expression. In summary, tumors positive for CXCR4 cytoplasmic staining are more likely to be positive for HER2 over-expression.

As an independent prognostic factor for breast cancer patients, EGFR is associated with a number of pathological characteristics of breast cancer. According to the results, EGFR expression is correlated with lymph node metastasis and histological grade (Table 1). Interestingly, during analysis, it was discovered that close to 70% of patients with high EGFR expression were CXCR4 and CCR7 positive as well. Spearmam's rank correlation analysis revealed that EGFR expression was significantly associated with CXCR4 cytoplasmic positivity and high CCR7 expression (P < 0.01; Table 3).

**Table 3:** Correlation of CXCR4, CCR7 and EGFR

Variable	Rho	P value
CXCR4 cytoplasmic and EGFR	0.255	<0.001**
CXCR4 nuclear and EGFR	0.046	0.515
CXCR4 cytoplasmic and CCR7	0.383	<0.001**
CXCR4 nuclear and CCR7	0.188	0.008**
CCR7 and EGFR	0.186	0.008**

The correlation between every two biomarkers was tested by Spearman's rank correlation test. *P < 0.05, **P < 0.01

### Concordance of CXCR4, CXCL12, CCR7, and CCL21 expression

After performing IHC staining for the two CXCL12 and CCL21 chemokines, it was revealed that these were correlated with one another (P = 0.017, Table 4), indicating a tendency towards co-expression of these molecules in tumors. Hence, the expression of their receptors, CXCR4 and CCR7 was likely to be tightly linked (P = .008; Table 4). No significant association was present between the expression of CXCR4 and CXCL12, nor between CCR7 and its chemokine ligand CCL21 (Table 4).

**Table 4:** Correlation of CXCR4, CCR7 and their ligands CXCL12, CCL21

Variable	Rho	P value
CXCR4 cytoplasmic and CXCL12	0.035	0.731
CCR7 and CCL21	0.017	0.863
CXCL12 and CCL21	0.238	0.017*

Correlation was tested by Spearman's rank correlation test. *P < 0.05

### CXCR4, CCR7, and EGFR demonstrate poor prognosis by survival analysis

**Figure 2:**
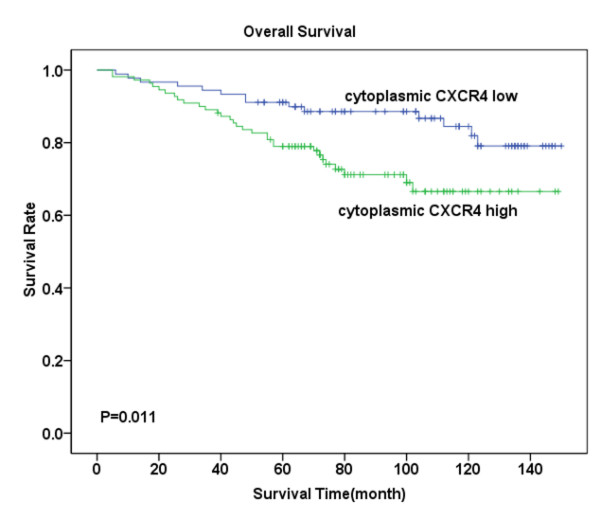
**Overall survival analysis for CXCR4 cytoplasmic expression**. Kaplan-Meier curves for overall survival (OS) in 110 patients with high expression of CXCR4 and 90 patients with low expression of CXCR4 in cytoplasm. Survival time sharply decreased in patients with high CXCR4 cytoplasmic expression, especially in the first five years, Meanwhile, survival of patients with low CXCR4 expression was merely moderately affected (P = 0.011).

**Figure 3:**
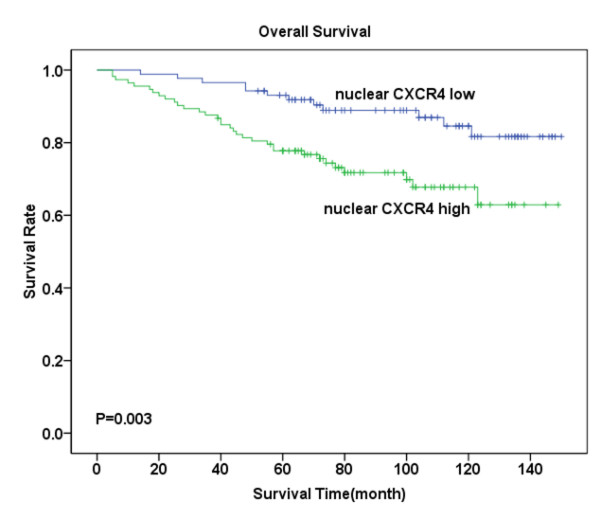
**Overall survival analysis for CXCR4 nuclear expression**. Kaplan-Meier curves for overall survival (OS) in 113 patients With high CXCR4 expression and 87 patients with low CXCR4 expression in the nucleus. Survival time sharply decreased in patients with high CXCR4 nuclear expression, especially in the first five years, when significantly compared with those exhibiting low expression (P = 0.003).

**Figure 4:**
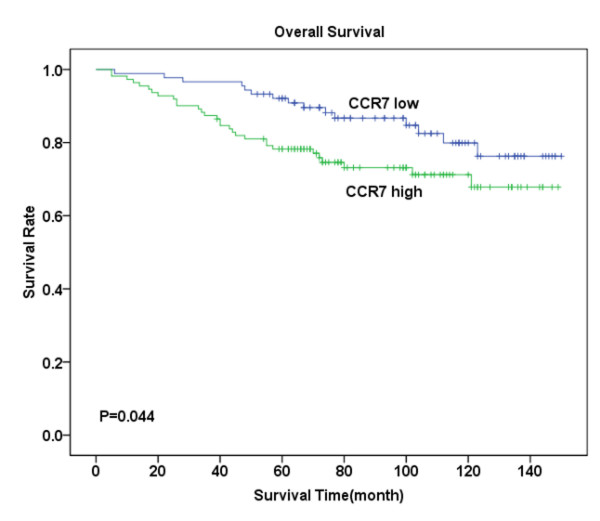
**Overall survival analysis for CCR7 expression**. Kaplan-Meier curves for overall survival (OS) in 111 patients with high CCR7 expression and 89 patients with low CCR7 expression in the cytoplasm. The difference between these two groups is not highly significant as determined by the log-rank test (P = 0.044). However, it can be observed from the curve that in the first five years, survival rate sharply decreased in patients with high CCR7 expression in the cytoplasm, while hardly any patient in the low expression group died during the first five years.

**Figure 5:**
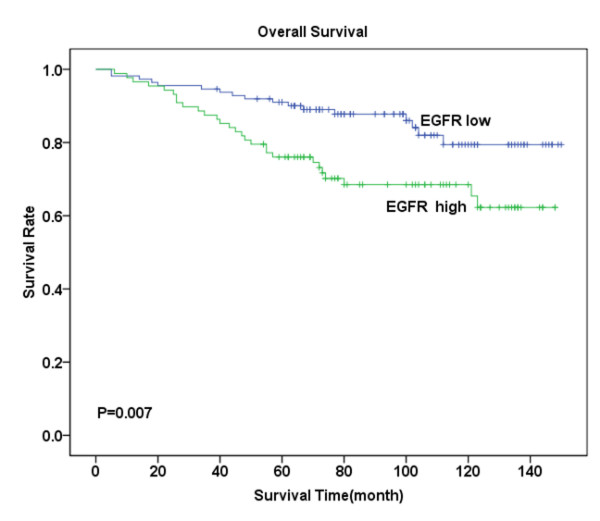
**Overall survival analysis for EGFR expression**. Kaplan-Meier curves for overall survival (OS) in 88 patient with high EGFR expression and 112 patients with low EGFR expression in the membrane and cytoplasm. Survival rate of patients with high EGFR expression was significantly low compared with those exhibiting low expression (P = 0.007).

Follow-up investigation revealed that the median survival time was 88 months (ranging from 5-150 months), within which 45 patients (22.5%) died because of breast cancer including 28 (28%) in the tumor with metastasis group and 17 (17%) in the non-metastasis group. Kaplan-Meier analysis revealed that patients suffering from high levels of CXCR4 expression- either in the cytoplasm or in the nucleus -had significantly lower OS compared with those with low CXCR4 expression (P = 0.011, Figure 2; P = 0.003, Figure 3). Similarly, high levels of CCR7 and EGFR expression revealed poor prognosis (P = 0.044, Figure 4; P = 0.007, Figure 5).

## Discussion

Recently, reports have demonstrated that chemokines and their receptors play critical roles in the development of cancer, including tumor cell growth, migration, and angiogenesis. Further, they influence the infiltration of immune cells in a tumor [8,9]. The mechanism of chemokines in malignant tumor metastasis may be reflected by the production of chemokine receptors by tumor cells, which respond to their homologous ligands (produced by certain organs) and migrate along the chemokine gradients to trigger specific organ metastasis [10]. Out of all the known chemokine receptors, breast cancer cells specifically express active CXCR4 and CCR7, the ligands of which are HCXCL12 and CCL21, respectively [2].

This study investigated a series of matched primary and lymph node metastasis breast cancer tumors to demonstrate whether the expression of the CXCR4 and CCR7 chemokine receptors, along with expression of EGFR, predicts increased risk of metastasis and mortality. Present data are consistent with those in previous reports describing a positive correlation between CXCR4 expression and lymph node metastasis in cases of non-small-cell lung cancer (NSCLC), nasopharyngeal cancer, colorectal cancer, and esophageal cancer [11-14]. Positive correlation has likewise been reported between CCR7 expression and lymph node metastasis in cases of NSCLC, breast, gastric, colorectal, esophageal, and thyroid cancer [15-20].

It has been demonstrated that the CXCR4/CXCL12 axis likewise induces chemotaxis and breast cancer cell migration. Since Muller reported that CXCR4-CXCL12 interaction governed the pattern of breast cancer metastasis in a mouse model, subsequent studies have been conducted in different tumors [2]. One study determined that the CXCR4 expression pattern correlated significantly with the degree of lymph node metastasis by investigating CXCR4 expression in 79 cases of invasive duct cancer (IDC) [21]. Su examined 85 cases of early breast carcinoma and learned that high cytoplasmic expression of CXCR4 is associated with axillary nodal metastasis [22]. In the prent study, CXCR4 was found to be present in both cytoplasm and nucleus of tumor cells, and cytoplasmic expression was associated with lymph node metastasis. This result is similar to that of certain studies [22-25], but is contrary to a handful of reports [26]. Further, CXC chemokine 12 (CXCL12, likewise known as stromal cell derived factor-1α, or SDF-1α) is expressed in the liver, lungs, brain, bone, and lymph nodes. on the other hand, CXCR4 is a membrane-bound G-protein-coupled receptor which, together with its ligand CXCL12, mediates inflammatory and tumor cell migration [27]. One study has also observed CXCR4 localization at the cytoplasm in leukocyte cell lines with enforced CXCR4 expression and CXCL12-induced polarization of CXCR4 to the edge of migrating leukocyte cells [25]. Hence, with regard to the effect of CXCL12, CXCR4 reactivity in the cytoplasm may reflect receptor internalization. This may be viewed as an activation state of CXCR4. Through immunohistochemistry, CXCL12 protein in the cytoplasm of tumor cells was located as well, and CXCL12 expression was observed to be higher in lymph node metastasis tumors than in primary tumors. This distinction in expression sites between chemokines and their receptors illustrates that CXCL12 attracts CXCR4 to certain metastatic sites along the concentration gradient.

Although nuclear CXCR4 is expressed in cancer cells, its function remains unclear. Spano demonstrated that nuclear CXCR4 expression represents a better outcome in patients afflicted with non-small-cell lung cancer [28]. However, in the present study, after over 10 years of follow-up observation conducted among 200 breast cancer patients, it was noted that high expression of both cytoplasmic and nucleus CXCR4 often indicated worse prognosis. Different localization patterns of chemokine receptors-whether nuclear or cytoplasmic-may have different levels of biological significance in cancer cells.

Similarly, the interaction between CCL21 and its CCR7 receptor plays a crucial role in lymphocytes homing to secondary lymphoid organs through lymphatic vessels. A study indicates that the hindrance of T cells homing to secondary lymphoid organs occurs because of the loss of CCL21 or the deletion of the CCR7 gene [29]. Hence, it is likely that the mechanism of CCL21 mediating migration of tumor cells to lymph nodes from primary site arising from its attraction to CCR7, which is highly expressed by primary tumors, is similar to the mechanism of the lymphocytes' homing effect. Results of this study revealed that 70% of primary breast cancer tissues and 77% of metastasis cancer cells in lymph nodes expressed CCR7. Further, there was a significant correlation between CCR7 expression and lymph node metastasis (p < 0.001); CCL21 was especially highly expressed in lymph nodes metastasis tumor cells (68%), which was not the case in primary tumor cells (P = 0.004).

Survival analysis revealed patients with highly expressed CCR7 are subject to a more undesirable prognosis compared with those who expressed low CCR7. Findings of this study coincide with those of other studies [7]. In view of all the evidences, there is reason to believe that the CCR7-CCL21 axis is a crucial factor in tumor lymph node metastasis. Moreover, as staining for CXCL12 and CCL21 (or CXCR4 and CCR7) was tightly linked in the group of primary tumors and lymph node metastasis tumors in this study, it is likely that a shared mechanism may account for variations in expression levels of both molecules in breast cancer. Coinciding with previous studies, it was demonstrated that levels of combined CCR7 and CXCR4 expression significantly correlated with lymph node metastatic status[16,17,30].

Recent studies and analyses conducted in the present study clearly indicate that EGFR expression serves as the strong prognostic factor in invasive breast cancer [23,31,32]. In this study, it was observed that patients with high EGFR expression are more prone to developing metastasis and possessing high grades of tumor, which are both important prognostic factors for breast cancer patients. Through survival analysis, it has been discovered that patients who highly express EGFR are subject to poor prognoses compared with those with low EGFR expression. Recent reports further suggest that CXCR4 expression can be up-regulated by HER-2/neu, which is required for HER2-mediated invasion in vitro and lung metastases in vivo [33]. Moreover, the result of the correlation between CXCR4, CCR7, EGFR, and HER-2/neu illustrates that the expression of chemokine receptors (CXCR4 and CCR7) is tightly associated with growth factors (EGFR and HER-2/neu). Based on this finding, it may be inferred that regulating growth factors may influence the expression of chemokine receptors, which may be helpful in identifying new pathways in breast cancer therapy.

This study was based on a small group of patients. However, it examined corresponding lymph nodes of each patient, and this has not been reported by other scholars to date. Although immunochemistry detection of the biomarkers may have certain limitations, it is a simple and widely utilized technique which can be carried out on routine paraffin-embedded tissues. By contrast, majority of new biological methods require specialized platforms and expertise that are considered impractical in routine pathological diagnosis.

## Conclusion

By examining the expression of chemokines and their receptors in both primary tumors and corresponding lymph node metastasis tumors, data indicate that chemokines and their receptors are differentially expressed in the primary and metastatic sites of breast cancer. Results reveal the significant association of CXCR4, CCR7, and EGFR with metastasis and poor prognosis. Further, the correlation between chemokine receptors and growth factors may provide a new method of understanding breast cancer metastasis and therapy, which are worthy of further study.

## Competing interests

The authors declare that they have no competing interests.

## Authors' contributions

Before submission, all authors read and approved the final manuscript. Among the authors, LYX designed the study, while JR collected the materials, performed all experiments, and drafted the manuscript. LJY conducted the statistical analysis and GQ accomplished construction of tissue microarray blocks. ZXL participated in the instruction of the experiment, while ST revised the manuscript critically to ensure important intellectual content. WJJ and LYX read and reviewed the sections, while, LJB and DQY performed follow-up observations on all patients. SBC provided the study concept and participated in its design and coordination.

## References

[B1] Hassan S Baccarelli A Salvucci O Basik M Plasma stromal cell derived factor-1: host derived marker predictive of distant metastasis in breast cancer Clin Cancer Res 2008 14 446 454 10.1158/1078-0432.CCR-07-1189 18223219

[B2] Müller A Homey B Soto H Ge N Catron D Buchanan ME McClanahan T Murphy E Yuan W Wagner SN Barrera JL Mohar A Verástegui E Zlotnik A Involvement of chemokine receptors in breast cancer metastasis Nature 2001 410 50 56 10.1038/35065016 11242036

[B3] Paget S The distribution of secondary growths in cancer of the breast Cancer Metastasis Rev 1989 8 98 101 2673568

[B4] Hassan S Ferrario C Saragovi U Quenneville L Gaboury L Baccarelli A Salvucci O Basik M The influence of tumor-host interactions in the stromal cell-derived factor-1/CXCR4 ligand/receptor axis in determining metastatic risk in breast cancer Am J Pathol 2009 175 66 73 10.2353/ajpath.2009.080948 19497995 PMC2708795

[B5] Cabioglu N Gong Y Islam R Broglio KR Sneige N Sahin A Gonzalez-Angulo AM Morandi P Bucana C Hortobagyi GN Cristofanilli M Expression of growth factor and chemokine receptors: new insights in the biology of inflammatory breast cancer Ann Oncol 2007 18 1021 1029 10.1093/annonc/mdm060 17351259

[B6] Cabioglu N Sahin AA Morandi P Meric-Bernstam F Islam R Lin HY Bucana CD Gonzalez-Angulo AM Hortobagyi GN Cristofanilli M Chemokine receptors in advanced breast cancer: differential expression in metastatic disease sites with diagnostic and therapeutic implications Ann Oncol 2009 20 1013 1019 10.1093/annonc/mdn740 19237480 PMC4318926

[B7] Mattern J Koanagi R Volm K Association of vascular endothelium growth factor expression with intratumoral microvessel density and tumor cell proliferation in human epidermoid lung cancer Br J Cancer 1996 73 931 934 8611409 10.1038/bjc.1996.166 PMC2074250

[B8] Zlotnik A Chemokines and cancer Int J Cancer 2006 119 2026 2029 10.1002/ijc.22024 16671092

[B9] Feng LY Ou ZL Wu FY Shen ZZ Shao ZM Involvement of a novel chemokine decoy receptor CCX-CKR in breast cancer growth, metastasis and patient survival Clin Cancer Res 2009 15 2962 2970 10.1158/1078-0432.CCR-08-2495 19383822

[B10] Wang J Seethala RR Zhang Q Gooding W van Waes C Hasegawa H Ferris RL Autocrine and paracrine chemokine receptor 7 activation in head and neck cancer: implications for therapy J Natl Cancer Inst 2008 100 502 512 10.1093/jnci/djn059 18364504

[B11] Na IK Scheibenbogen C Adam C Stroux A Ghadjar P Thiel E Keilholz U Coupland SE Nuclear expression of CXCR4 in tumor cells of non-small cell lung cancer is correlated with lymph node metastasis Hum Pathol 2008 39 1751 1755 10.1016/j.humpath.2008.04.017 18701133

[B12] Hu J Deng X Bian X Li G Tong Y Li Y Wang Q Xin R He X Zhou G Xie P Li Y Wang JM Cao Y The expression of functional chemokine receptor CXCR4 is associated with the metastatic potential of human nasopharyngeal carcinoma Clin Cancer Res 2005 11 4658 4665 10.1158/1078-0432.CCR-04-1798 16000558

[B13] Yoshitake N Fukui H Yamagishi H Sekikawa A Fujii S Tomita S Ichikawa K Imura J Hiraishi H Fujimori T Expression of SDF-1 alpha and nuclear CXCR4 predicts lymph node metastasis in colorectal cancer Br J Cancer 2008 98 1682 1689 10.1038/sj.bjc.6604363 18443596 PMC2391124

[B14] Gockel I Schimanski CC Heinrich C Wehler T Frerichs K Drescher D von Langsdorff C Domeyer M Biesterfeld S Galle PR Junginger T Moehler M Expression of chemokine receptor CXCR4 in esophageal squamous cell and adenocarcinoma BMC Cancer 2006 6 290 296 10.1186/1471-2407-6-290 17176471 PMC1766934

[B15] Takanami I Overexpression of CCR7 mRNA in nonsmall cell lung cancer: correlation with lymph node metastasis Int J Cancer 2003 105 186 189 10.1002/ijc.11063 12673677

[B16] Cabioglu N Yazici MS Arun B Broglio KR Hortobagyi GN Price JE Sahin A CCR7 and CXCR4 as novel biomarkers predicting axillary lymph node metastasis in T1 breast cancer Clin Cancer Res 2005 11 5686 5693 10.1158/1078-0432.CCR-05-0014 16115904

[B17] Arigami T Natsugoe S Uenosono Y Yanagita S Arima H Hirata M Ishigami S Aikou T CCR7 and CXCR4 expression predicts lymph node status including micrometastasis in gastric cancer Int J Oncol 2009 35 19 24 10.3892/ijo_00000308 19513547

[B18] Akishima-Fukasawa Y Nakanishi Y Ino Y Moriya Y Kanai Y Hirohashi S Prognostic significance of CXCL12 expression in patients with colorectal carcinoma Am J Clin Pathol 2009 132 202 210 10.1309/AJCPK35VZJEWCUTL 19605814

[B19] Ding Y Shimada Y Maeda M Kawabe A Kaganoi J Komoto I Hashimoto Y Miyake M Hashida H Imamura M Association of CC chemokine receptor 7 with lymph node metastasis of esophageal squamous cell carcinoma Clin Cancer Res 2003 9 3406 3412 12960129

[B20] Sancho M Vieira JM Casalou C Mesquita M Pereira T Cavaco BM Dias S Leite V Expression and function of the chemokine receptor CCR7 in thyroid carcinomas J Endocrinol 2006 191 229 238 10.1677/joe.1.06688 17065406

[B21] Kato M Kitayama J Kazama S Nagawa H Expression pattern of CXC chemokine receptor-4 is correlated with lymph node metastasis in human invasive ductal carcinoma Breast Cancer Res 2003 5 R144 R150 10.1186/bcr627 12927045 PMC314431

[B22] Su YC Wu MT Huang CJ Hou MF Yang SF Chai CY Expression of CXCR4 is associated with axillary lymph node status in patients with early breast cancer Breast 2006 15 533 539 10.1016/j.breast.2005.08.034 16239110

[B23] Blot E Laberge-Le Couteulx S Jamali H Cornic M Guillemet C Duval C Hellot MF Pille JY Picquenot JM Veyret C CXCR4 membrane expression in node-negative breast cancer Breast J 2008 14 268 274 10.1111/j.1524-4741.2008.00573.x 18373506

[B24] Salvucci O Bouchard A Baccarelli A Deschênes J Sauter G Simon R Bianchi R Basik M The role of CXCR4 receptor expression in breast cancer: a large tissue microarray study Breast Cancer Res Treat 2006 97 275 283 10.1007/s10549-005-9121-8 16344916

[B25] Yasuoka H Tsujimoto M Yoshidome K Nakahara M Kodama R Sanke T Nakamura Y Cytoplasmic CXCR4 expression in breast cancer: induction by nitric oxide and correlation with lymph node metastasis and poor prognosis BMC Cancer 2008 8 340 349 10.1186/1471-2407-8-340 19025611 PMC2642845

[B26] Woo SU Bae JW Kim CH Lee JB Koo BW A significant correlation between nuclear CXCR4 expression and axillary lymph node metastasis in hormonal receptor negative breast cancer Ann Surg Oncol 2007 15 281 285 10.1245/s10434-007-9595-1 17763975

[B27] Tarasova NI Stauber RH Michejda CJ Spontaneous and ligandinduced trafficking of CXC-chemokine receptor 4 J Biol Chem 1998 273 15883 15886 10.1074/jbc.273.26.15883 9632631

[B28] Spano JP Andre F Morat L Sabatier L Besse B Combadiere C Deterre P Martin A Azorin J Valeyre D Khayat D Le Chevalier T Soria JC Chemokine receptor CXCR4 and early-stage non-small cell lung cancer: pattern of expression and correlation with outcome Ann Oncol 2004 15 613 617 10.1093/annonc/mdh136 15033669

[B29] Gunn MD Kyuwa S Tam C Kakiuchi T Matsuzawa A Williams LT Nakano H Mice lacking expression of secondary lymphoid organ chemokine have defects in lymphocyte homing and dendritic cell localization J Exp Med 1999 189 451 460 10.1084/jem.189.3.451 9927507 PMC2192914

[B30] Kodama J Hasengaowa Kusumoto T Seki N Matsuo T Ojima Y Nakamura K Hongo A Hiramatsu Y Association of CXCR4 and CCR7 chemokine receptor expression and lymph node metastasis in human cervical cancer Ann Oncol 2007 18 70 76 10.1093/annonc/mdl342 17032700

[B31] Singh S Singh UP Grizzle WE Lillard JW Jr CXCL12-CXCR4 interactions modulate prostate cancer cell migration, metalloproteinase expression and invasion Lab Invest 2004 84 1666 1676 10.1038/labinvest.3700181 15467730

[B32] Buchholz TA Tu X Ang KK Esteva FJ Kuerer HM Pusztai L Cristofanilli M Singletary SE Hortobagyi GN Sahin AA Epidermal growth factor receptor expression correlates with poor survival in patients who have breast carcinoma treated with doxorubicin-based neoadjuvant chemotherapy Cancer 2005 104 676 681 10.1002/cncr.21217 15981280

[B33] Li YM Pan Y Wei Y Cheng X Zhou BP Tan M Zhou X Xia W Hortobagyi GN Yu D Hung MC Upregulation of CXCR4 is essential for HER2 mediated tumor metastasis Cancer Cell 2004 6 459 469 10.1016/j.ccr.2004.09.027 15542430

